# Association between trunk pain and lower extremity pain among youth soccer players: a cross-sectional study

**DOI:** 10.1186/s13102-018-0102-8

**Published:** 2018-07-06

**Authors:** Yasuhito Sogi, Yoshihiro Hagiwara, Yutaka Yabe, Takuya Sekiguchi, Haruki Momma, Masahiro Tsuchiya, Kaoru Kuroki, Kenji Kanazawa, Masashi Koide, Nobuyuki Itaya, Shinichiro Yoshida, Toshihisa Yano, Eiji Itoi, Ryoichi Nagatomi

**Affiliations:** 1 Department of Orthopaedic Surgery, Tohoku University School of Medicine, 1-1 Seiryo-machi, Aoba-ku, Sendai, 980-8574 Japan; 2 Department of Medicine and Science in Sports and Exercise, Tohoku University School of Medicine, 2-1 Seiryo-machi, Aoba-ku, Sendai, 980-8575 Japan; 3 Division of Biomedical Engineering for Health and Welfare, Tohoku University Graduate School of Biomedical Engineering, 2-1 Seiryo-machi, Aoba-ku, Sendai, 980-8575 Japan; 4 Department of Nursing, Faculty of Health Science, Tohoku Fukushi University, 1-8-1 Kunimi, Aoba-ku, Sendai, 981-8522 Japan; 5 Department of Rehabilitation, Tohoku Fukushi University, 1-8-1 Kunimi, Aoba-ku, Sendai, 981-8522 Japan

**Keywords:** Knee pain, Ankle pain, Trunk, Lower extremity, Youth soccer, Epidemiological study

## Abstract

**Background:**

Soccer is a high-intensity sport with a high injury rate. Among youth soccer players, lower extremity pain is a major problem that could be associated with trunk function. This study investigated the association between lower extremity pain and trunk pain among youth soccer players.

**Methods:**

A cross-sectional study involving youth soccer players participating in the Miyagi Amateur Sports Association was conducted using a self-reported questionnaire. A multiple logistic regression analysis was used to examine the association between trunk pain and lower extremity pain. Covariates were sex, age, body mass index, height increase, number of days of training per week, practice time per day on weekdays or weekends, competition levels, frequency of participation in games, and previous injuries**.**

**Results:**

The final study population comprised 1139 youth soccer players (age, 6–15 years; male, 94.2%). Lower extremity pain with concomitant trunk pain occurred in 61.8% (42/68). Trunk pain was significantly associated with lower extremity pain (adjusted odds ratio [OR], 6.82; 95% confidence interval [CI], 3.99–11.67). Back pain and hip pain were significantly associated with knee pain (adjusted OR [95% CI]: 7.63 [3.70–15.76] and 3.84 [1.89–7.83], respectively), ankle pain (adjusted OR [95% CI]: 9.03 [4.42–18.44] and 5.43 [2.77–10.62], respectively), and both knee and ankle pain (adjusted OR [95% CI]: 13.67 [6.01–31.09] and 5.98 [2.56–13.97], respectively).

**Conclusions:**

Trunk pain was associated with lower extremity pain among youth soccer players. Clinicians and coaches should consider comorbidities while treating those players.

## Background

Lower extremity pain among cLhildren and adults is a major health problem [1]. The prevalence of lower limb pain in children and adolescents has been reported to be 29–40%, and it often involves chronic or recurrent pain [2, 3]. In a study performed in northern Finland, knee pain frequency was 18.5% among adolescents and 3.9% among children, and 56% of cases were associated with sports activities among adolescents and children [4]. The lower extremities are the most popular injury sites and account for more than half of all sports injuries [5]. Witvrouw et al. reported that the most common cause of knee pain among youth athletes was patellofemoral disorder, which is caused by activities such as running, squatting, and jumping [6]. Ankle ligament injuries and anterior cruciate ligament (ACL) injuries are also common among soccer and basketball players [5]. Furthermore, non-traumatic pain caused by overuse, such as Osgood-Schlatter disease and Sever’s disease, is common in youth and sometimes results in long-term disability, leading to the inability to participate in sports [7–9]. Increases in sports specialization as well as sports-specific skills have occurred among youth players, which could result in several musculoskeletal disorders [10].

In Japan, soccer is popular; therefore, the number of players is increasing [11]. Soccer is a high-intensity sport that requires frequent changes in movements, velocity, and directions [12]. These characteristics can easily induce acute lower extremity injuries or non-traumatic overuse injuries in youth players [7, 8, 13]. The youth injury rate has been reported to be 5.6 to 28.3 per 1000 player hours [14, 15], which is higher than that for other sports [16]. According to a cross-sectional study of 1162 youth soccer players in Japan, the rate of lower extremity pain is higher than that for other body parts [17]. Most pain is not severe and resolves within a few weeks [18]; however, some players experience persistent chronic pain that causes them to stop participating in sports activities [18–20].

To prevent injuries related to soccer, the FIFA 11+ warm-up program is used [21]. This program includes exercises for hip and trunk static/dynamic balance, strength, agility, and plyometrics, and it can help reduce the incidence of lower extremity injuries [22]. Previous studies revealed an association between lower extremity injuries and trunk function in adults [23, 24]. In biomechanical studies, trunk muscle strength and stability were important and protected the lower extremity position against unexpected force [23, 24]. Additionally, warm-up programs that focused on trunk function could reduce the incidence of non-traumatic lower extremity injuries [21]. Some studies involving adults showed that trunk dysfunction, including hypomobility of the lumber spine and hip, was associated with low back pain [25, 26]. Therefore, we supposed that trunk symptoms could be associated with lower extremity pain. It is important to know this association to prevent lower extremity pain. The purpose of this study was to investigate the association between trunk pain and lower extremity pain among youth soccer players.

## Methods

### Study design and participants

**Fig. 1:**
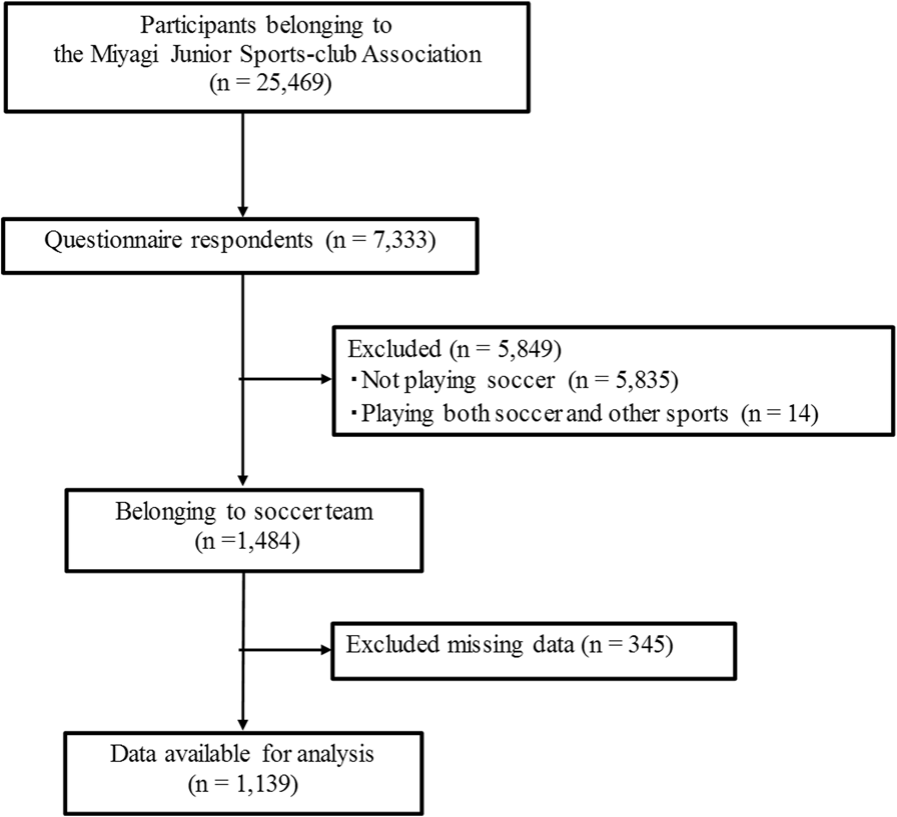
Study flowchart of this study

Study participants were school-aged athletes participating in the Miyagi Amateur Sports Association in Japan. It was established with the aim of promoting youth health through various sports (soccer, baseball, basketball, volleyball, judo, kendo, karate, athletics, skiing, badminton, swimming, etc.). Inclusion criteria of this study were youth athletes aged 6–15 years who only played soccer, whereas exclusion criteria were participants who did not agree to participate, who did not provide informed consent, or who had missing data. The organization has mailing address information for 25,469 registered athletes and their parents. A self-administered questionnaire and an informed consent document were mailed to all of them in October 2014. Among them, 7333 athletes submitted both written informed consent and the questionnaire by the end of December 2014 (response rate, 28.8%; 7333/25,469). Those who did not play soccer (*n* = 5835) and those who played soccer and other sports (*n* = 14) were excluded from this study. Moreover, we excluded 345 participants who had not answered all the questions of the questionnaire. The final study population comprised 1139 youth soccer players (Fig. 1). The study protocol was reviewed and approved by the Ethics Committee on Research of Human Subjects at the Tohoku University Graduate School of Medicine (approval number: 2013–1-564).

### Dependent and independent variables

Lower extremity pain was assessed as the dependent variable and trunk pain was assessed as the independent variable. The pain location was assessed using a self-reported questionnaire. The question regarding the presence of pain was, “Do you have pain in any parts of your body now? If yes, please check the following parts (multiple choices were allowed).” The choices were as follows: “(1) back, (2) low back, (3) buttocks, (4) hips, (5) knees, (6) ankle-foot areas, (7) shoulders, (8) elbows, and (9) wrists/hands.” These anatomical areas were indicated by a drawing [27]. Back pain, low back pain, and buttock pain were summarized as back pain according to a previous report [28]. Lower extremity pain was defined as pain in the knee and/or ankle-foot area. Hip pain was classified as trunk pain according to the previous study, and trunk pain was defined as pain in the back and/or hip. [24].

### Covariates

The following were included in the analyses as covariates: sex, age, body mass index (BMI), height increase (cm/year), number of days of training per week, practice time per day on weekdays or weekends, competition levels, frequency of participation in games, and previous injuries. Height increase was assessed through the following question: “How much taller did you grow in the last year?” The following continuous variables were divided into categories according to distribution or clinical significance when necessary: age (6–10 years or 11–15 years); BMI (< 18.5, 18.5 to < 25, or ≥ 25); number of days of training per week (< 3 or ≥ 3 days); amount of practice time per day on weekdays (< 2 or ≥ 2 h); and amount of practice time per day on weekends (< 3 or ≥ 3 h).

Pre-coded questions included competition levels (“national competition,” “Tohoku distinct competition,” “prefectural competition,” “local competition,” or “only recreation”), frequency of participation in games (“often,” “sometimes,” “seldom,” or “never”), and previous injuries (“absence,” “presence,” or “unknown”). Competition levels and frequency of participation in games were categorized into two groups according to the distribution and clinical significance (competition levels: “high; prefectural competition or more” and “low; local competition or only recreation” and frequency: “frequently; often” and “not frequently; sometimes or less”).

### Statistical analysis

Continuous variables were presented as medians with interquartile range (IQR); categorical variables were presented as numbers and percentage (%). First, we performed crude and multiple logistic regression analyses to examine the association of trunk pain with lower extremity pain. The odds ratio (OR) and 95% confidence intervals (95% CI) for the presence of lower extremity pain were calculated after simultaneous adjustment for potential covariates. Second, multinomial logistic regressions analyses were performed to evaluate the association of back pain or hip pain with knee pain, ankle pain, or both, respectively. The ORs and 95% CI for the presence of knee pain, ankle pain, or both were calculated after simultaneous adjustments for potential covariates. Covariates included sex (female or male), age (6–10 years or 11–15 years), BMI (< 18.5, 18.5 to < 25, or ≥ 25), height increase (continuous variables), number of days participating in training per week (< 3 or ≥ 3 days), amount of practice time on weekdays per week (< 2 or ≥ 2 h), amount of practice time on weekends per week (< 3 or ≥ 3 h), competition levels (low or high), frequency of participation in games (frequently or not frequently), and history of injuries (absence, presence, or unknown). To check potential collinearity between independent variables and covariates, we calculated the variance inflation factors (VIFs). All statistical analyses were performed using SPSS version 24.0 (SPSS Japan Inc., Tokyo, Japan). All tests were two-tailed, and *p* < 0.05 was considered statistically significant.

## Results

Baseline characteristics of the participants are shown in Table 1. The median (IQR) age of the participants was 11 years (9–12), and the median BMI was 16.6 (15.5–18.1). VIFs were low, ranging from 1.01 to 1.20, suggesting that collinearity among the adjusted variables was not a significant problem. A significantly high rate of lower extremity pain was observed in the older group (11–15 years) compared to the younger group (6–10 years) (OR [95% CI]: 1.65 [1.22–2.24]). The presence of a previous injury was significantly associated with a lower extremity injury (OR [95% CI]: 3.03 [2.16–4.24]).

**Table 1:** Baseline characteristics of participants

			Pain sites		
Characteristics	Median (IQR)	n (%)	Back (*n* = 36)	Hip (*n* = 40)	Trunk (*n* = 68)
Sex					
Female		66 (5.8)	2 (5.6)	1 (2.5)	3 (4.4)
Male		1073 (94.2)	34 (94.4)	39 (97.5)	65 (95.6)
Age (years)	11.0 (9.0 12.0)				
6–10		564 (49.5)	7 (19.4)	7 (17.5)	13 (19.1)
11–15		575 (50.5)	29 (80.6)	33 (82.5)	55 (80.9)
BMI (kg/m2)	16.6 (15.5 18.1)				
< 18.5		906 (79.5)	26 (72.2)	29 (72.5)	48 (70.6)
18.5 to < 25		220 (19.3)	9 (25.0)	11 (27.5)	19 (27.9)
≥ 25		13 (1.1)	1 (2.8)	0 (0.0)	1 (1.5)
Height increase (per year)	5.0 (4.0 7.0)		5.0 (3.0 9.5)	5.0 (4.0 8.7)	5.0 (3.5 8.7)
Number of days of training/week (days)	3.0 (2.0 4.0)				
< 3		368 (32.3)	12 (33.3)	9 (22.5)	18 (26.5)
≥ 3		771 (67.7)	24 (66.7)	31 (77.5)	50 (73.5)
Practice time/day on weekdays (hr)	2.0 (1.5 2.0)				
< 2		392 (34.4)	12 (33.3)	13 (32.5)	22 (32.4)
≥ 2		747 (65.6)	24 (66.7)	27 (67.5)	46 (67.6)
Practice time/day on weekends (hr)	3.0 (2.0 3.0)				
< 3		364 (32.0)	9 (25.0)	10 (25.0)	17 (25.0)
≥ 3		775 (68.0)	27 (75.0)	30 (75.0)	51 (75.0)
Competitive levels					
Low		599 (52.6)	17 (47.2)	16 (40.0)	30 (44.1)
High		540 (47.4)	19 (52.8)	24 (60.0)	38 (55.9)
Frequency of participation in games					
Frequently		725 (63.7)	26 (72.2)	27 (67.5)	48 (70.6)
Not frequently		414 (36.3)	10 (27.8)	13 (32.5)	20 (29.4)
Previous injuries					
Absence		532 (46.7)	10 (27.8)	7 (17.5)	15 (22.1)
Presence		541 (47.5)	25 (69.4)	30 (75.0)	49 (72.1)
Unknown		66 (5.8)	1 (2.8)	3 (7.5)	4 (5.9)

The prevalence of pain in each body part was as follows: 9.4% (*n* = 107) in the knee, 13.3% (*n* = 152) in the ankle, 18.7% (*n* = 213) in the lower extremity, 3.2% (*n* = 36) in the back, 3.5% (*n* = 40) in the hip, and 6.0% (*n* = 68) in the trunk. The prevalence of lower extremity pain among participants with trunk pain was 19.7% (*n* = 42). The crude and adjusted ORs (95% CI) for the presence of lower extremity pain among the categories of trunk pain are shown in Table 2. The presence of trunk pain was significantly associated with the presence of lower extremity pain regardless of the adjustment for potential confounding factors (adjusted OR, 6.82 [3.99–11.67]). The crude and adjusted ORs (95% CI) for the presence of knee pain, ankle pain, or both among the participants with back pain or hip pain are shown in Table 3. Back pain and hip pain were significantly associated with knee pain (adjusted OR [95% CI]: back pain: 7.63 [3.70–15.76], *p* < 0.001; hip pain: 3.84 [1.89–7.83], *p* < 0.001), ankle pain (adjusted OR [95% CI]: back pain: 9.03 [4.42–18.44], p < 0.001; hip pain: 5.43 [2.77–10.62], *p* < 0.001), and both knee pain and ankle pain (adjusted OR [95% CI]: back pain: OR [95% CI]: 13.67 [6.01–31.09], *p* < 0.001; hip pain: 5.98 [2.56–13.97], *p* < 0.001).

**Table 2:** Association between trunk pain and lower extremity pain among youth soccer players

	Trunk pain		*P* value
	absence (*n* = 1071)	presence (*n* = 68)	
Lower extremity pain (*n* = 213)			
N (%)	171 (16.0)	42 (61.8)	
OR (95% CI)a	1.00	8.50 (5.08–14.24)	< 0.001
Adjusted OR (95% CI)b	1.00	6.82 (3.99–11.67)	< 0.001

^a^Crude model

^b^Adjusted for sex (female or male), age (6–10 years or 11–15 years), BMI (< 18.5, 18.5 to < 25, or ≥ 25), height increase (continuous variable), number of days of training time/week (< 3 or ≥ 3), practice time/day on weekdays (< 2 or ≥ 2 h), practice time/day on weekends (< 3 or ≥ 3 h), competitive levels (low or high), frequency of participation in games (frequently or not), and previous injuries (absence, presence or unknown)

*OR* Odds Ratio, *CI* Confidence Intervals

**Table 3:** Back pain and hip pain associated with knee pain and ankle pain among youth soccer players

Pain sites	Back pain		*P* value	Hip pain		*P* value
	absence (*n* = 1,103)	presence (*n* = 36)		absence (*n* = 1,099)	presence (*n* = 40)	
Knee pain (n = 107)						
OR (95% CI)^a^	1.00	10.07 (5.06-20.06)	<0.001	1.00	5.83 (2.94-11.54)	<0.001
Adjusted OR (95% CI)^b^	1.00	7.63 (3.70-15.76)	<0.001	1.00	3.84 (1.89-7.83)	<0.001
Ankle pain (n = 152)						
OR (95% CI)^a^	1.00	9.20 (4.65-18.19)	<0.001	1.00	6.57 (3.44-12.54)	<0.001
Adjusted OR (95% CI)^b^	1.00	9.03 (4.42-18.44)	<0.001	1.00	5.43 (2.77-10.62)	<0.001
Both knee pain and ankle pain (n = 46)						
OR (95% CI)^a^	1.00	15.72 (7.26-34.04)	<0.001	1.00	8.33 (3.70-18.76)	<0.001
Adjusted OR (95% CI)^b^	1.00	13.67 (6.01-31.09)	<0.001	1.00	5.98 (2.56-13.97)	<0.001

Reference category: Neither knee nor ankle pain

*OR* Odds Ratio, *CI* Confidence Intervals

^a^ Crude model

^b^ Adjusted for sex (female or male), age (6-10 years or 11-15 years), BMI (<18.5, 18.5 to <25, or ≥25), height increase (continuous variable), number of days of training time/week (<3 or ≥3), practice time/day on weekdays (<2 or ≥2 hrs), practice time/day on weekends (<3 or ≥3 hrs), competitive levels (low or high), frequency of participation in games (frequently or not), and previous injuries (absence, presence or unknown)

## Discussion

Our findings indicated that trunk pain was significantly associated with lower extremity pain among youth soccer players. Either back pain or hip pain was also associated with knee pain, ankle pain, or both.

The major traumatic injuries among youth soccer players are ankle sprains and knee ligament injuries, and these injuries occur regardless of contact or non-contact situations. During contact sports, these injuries are likely to be caused by player-to-player contact. However, for soccer, which is usually recognized as a contact sport, the incidence rate of non-contact ACL injuries is 26–59%, and some studies reported that this rate is higher than that for contact ACL injuries. This is because soccer players perform several maneuvers such as cutting, landing, and stopping [12, 29, 30]. Non-contact injuries are significantly associated with neuromuscular functions [31, 32]. Precise neural control of the muscles optimizes skill and movement, and its deficit leads to injuries of whole body sites, including the upper extremities and trunk [24, 33]. In addition, overuse injuries are also concomitant with whole body issues such as joint laxity and muscle tightness and asymmetry [18, 34]. These traumatic and overuse injuries are thought to be associated with general condition issues. Sekiguchi et al. reported that knee pain and low back pain among youth baseball players are associated with upper extremity pain due to a disturbance in kinetic chains [27]. Zazulak et al. reported that a history of low back pain could be a predictor of knee ligament injury in adults [24]. Luis et al. showed an association between foot arch posture and shoulder and elbow injuries [35]. Our results indicated that back pain or hip pain was associated with knee pain and/or ankle pain, corresponding with previous reports. To understand lower extremity injuries, whole bodies and injured sites should be the focus.

Some previous studies of adult athletes reported an association between lower extremity injuries and trunk function [23, 24, 28]. Among collegiate athletes, patellofemoral pain was associated with weakness of the hip abductor and external rotation strength [24]. Furthermore, muscle strength dysfunction was associated with ACL injuries that occurred when the knee was abducted and loaded with external force to the femur [23, 36]. Ankle sprains were also associated with weakness of the hip abductor muscle and less range of motion of plantar flexion [37]. Rassi et al. analyzed motions of the spine and hip during kicking a ball and reported that hyperextension and hyperflexion of the spine and hip were required [38]. Several studies have shown that people with low back pain tend to have hypomobility of the lumber spine and hip [25, 26]. Youth soccer players with trunk pain were assumed to have hypomobility of the trunk. Stability and strength of the trunk affect proper gait, foot posture, and knee loading because the trunk activities precede lower extremity activities, which are reduced due to hypomobility of the trunk [37, 39, 40]. Trunk pain can lead to abnormal lower extremity kinematics in youth soccer players, and these are assumed to cause lower extremity injuries and pain.

Numerous studies of the prevention of injuries have been reported, and they have focused on static and dynamic stability, core-focused strength, plyometrics, and agility [22, 41]. Some prevention programs are effective and others fail due to uncertain compliance [12, 42, 43]. Steffan et al. reported that they could not show significant effectiveness of a warm-up program for female soccer players [21]. However, during their prospective study, they reported that coaches who are motivated to prevent injuries by using the FIFA 11+ program could improve players’ physical functions and reduce the incidence of injuries among female soccer players [31]. These studies have suggested that decreased incidences of injuries are achieved by good adherence to the program. Subjective symptoms are noticeable by both players and coaches. This study revealed the association between lower extremity pain and trunk pain, which suggested that attention should be focused on pain in the trunk and lower extremities.

This study had several limitations. First, the study was cross-sectional, and reverse causality could not be ruled out. Second, we assessed pain only with self-reported survey responses of youth players. Severity and duration of the pain were not assessed, and the pathology of the pain was not clear. In addition, variables included only physical factors, not psychosocial considerations. Third, although the participants were youth players, we did not assess the reliability and validity of the questionnaire used for our study. Further prospective studies are needed to resolve these issues.

## Conclusion

Among youth soccer players in Japan, trunk pain was associated with lower extremity pain. Clinicians and coaches should consider comorbidities while treating those players.

## Abbreviations

ACL: Anterior cruciate ligament
VIFs: Variance inflation factors
